# Survival bias and drug interaction can attenuate cross-sectional case-control comparisons of genes with health outcomes. An example of the kinesin-like protein 6 (*KIF6*) Trp719Arg polymorphism and coronary heart disease

**DOI:** 10.1186/1471-2350-12-42

**Published:** 2011-03-24

**Authors:** Paul Williams, Lakshmana Pendyala, Robert Superko

**Affiliations:** 1 Celera, 1401 Harbor Bay Parkway, Alameda, CA 94502, USA; 2 University of Louisville, 550 South Jackson Street, Louisville, KY 40202, USA

## Abstract

**Background:**

Case-control studies typically exclude fatal endpoints from the case set, which we hypothesize will substantially underestimate risk if survival is genotype-dependent. The loss of fatal cases is particularly nontrivial for studies of coronary heart disease (CHD) because of significantly reduced survival (34% one-year fatality following a coronary attack). A case in point is the *KIF6 *Trp719Arg polymorphism (rs20455). Whereas six prospective studies have shown that carriers of the *KIF6 *Trp719Arg risk allele have 20% to 50% greater CHD risk than non-carriers, several cross-sectional case-control studies failed to show that carrier status is related to CHD. Computer simulations were therefore employed to assess the impact of the loss of fatal events on gene associations in cross-sectional case-control studies, using *KIF6 *Trp719Arg as an example.

**Results:**

Ten replicates of 1,000,000 observations each were generated reflecting Canadian demographics. Cardiovascular disease (CVD) risks were assigned by the Framingham equation and events distributed among *KIF6 *Trp719Arg genotypes according to published prospective studies. Logistic regression analysis was used to estimate odds ratios between *KIF6 *genotypes. Results were examined for 33%, 41.5%, and 50% fatality rates for incident CVD.

In the absence of any difference in percent fatalities between genotypes, the odds ratios (carriers vs. noncarriers) were unaffected by survival bias, otherwise the odds ratios were increasingly attenuated as the disparity between fatality rates increased between genotypes. Additional simulations demonstrated that statin usage, shown in four clinical trials to substantially reduce the excess CHD risk in the *KIF6 *719Arg variant, should also attenuate the *KIF6 *719Arg odds ratio in case-control studies.

**Conclusions:**

These computer simulations show that exclusions of prior CHD fatalities attenuate odds ratios of case-control studies in proportion to the difference in the percent fatalities between genotypes. Disproportionate CHD survival for *KIF6 *Trip719Arg carriers is suggested by their 50% greater risk for recurrent myocardial infarction. This, and the attenuation of *KIF6 *719Arg carrier risk with statin use, may explain the genotype's weak association with CHD in cross-sectional case-control studies. The results may be relevant to the underestimation of risk in cross-sectional case-control studies of other genetic CHD-risk factors affecting survival.

## Background

Case-control comparisons are more commonly employed for assessing gene associations than prospective cohort studies [1,2]. Their popularity arises from two important considerations: 1) statistical power is often limited by the number of cases, and 2) genetic investigations require no consideration of the sequence of cause and effect. In addition to their being cost-effective, case-control studies may be the only reasonable approach to study rare diseases [3]. Discussions of the advantages and disadvantages of case-control studies in the context of genetic studies, and the advantages of prospective cohort studies, have been discussed in several reviews [2,4].

Selection bias refers to subject composition affecting genotype-phenotype associations. For example, non-representative sample of the target population can affect the generalizability of genetic association studies, even if the biases do not differ between genotypes. More importantly with respect to genetic studies are sampling biases that distort genotype-phenotype associations. Such distortions may be less likely to occur in prospective cohort studies than case-control studies [2].

The potential for selection bias is an important limitation of cross-sectional case-control comparisons. Prospective studies (e.g., prospective cohorts and nested control designs) can avoid selection biases, including selection bias due to case fatalities, which can be substantial. For example, the American Heart Association reported that approximately 34% of those who experience a coronary attack in a given year die from it, as do 15% of those experiencing myocardial infarctions [5]. Thirty-six percent of men and 47% of women die within 5 years of their first myocardial infarction [5]. Prior history of myocardial infarction increases the risk for subsequent sudden death by four- to six-fold [5]. Depending on sex and clinical outcome, those who survive the acute stage of a myocardial infarction have 1.5- to 15-fold increased risk of illness and death vis-à-vis the general population [5]. These fatal events will be included among cases in prospective study designs, but missing in cross-sectional case-control comparisons.

A case in point is the kinesin-like protein 6 (*KIF6*) Trp719Arg polymorphism (rs20455). Table 1 summarizes the findings of seven prospective studies of the *KIF6 *Trp719Arg polymorphism in relation to CHD, including four in the absence of statin use: 1) the nested case-control comparison of placebo-treated West of Scotland Coronary Prevention Study (WOSCOPS) patients [6]; 2) the prospective follow-up of the placebo-treated patients in the Cholesterol and Recurrent Events (CARE) secondary prevention trial [7]; 3) the prospective follow-up of placebo-treated PROSPER (PROspective Study of Pravastatin in the Elderly at Risk) elderly patients with pre-existing vascular disease [8]; and 4) prospective follow-up of the Heart Protection Study (HPS) [9]. Taken together, WOSCOPS and CARE showed prospectively that *KIF6 *719Arg increased primary and secondary CHD risk by about 50% in statin-untreated subjects with moderate to high LDL-cholesterol. This risk estimate is somewhat greater than the 28% increased risk for fatal and nonfatal cardiovascular events for *KIF6 *719Arg carriers (P = 0.07, where carriers have at least one copy of the risk allele) in PROSPER [8], and the 17% increased risk for major coronary events in HPS (P = 0.2) [9]. Statin use has been shown to mitigate or eliminate the higher CHD risk of *KIF6 *719Arg carriers [6,8,10], and three other prospective studies of Table 1 did not control for statin use [11-14]. These include the Women's Health Study (WHS) [11], the Cardiovascular Health Study (CHS) [12], and the Atherosclerosis Risk in Communities study (ARIC) [13,14]. Nevertheless, all three assign greater CHD risk to *KIF6 *719Arg carriers [11,12] or homozygotes [13,14].

**Table 1:** Studies of *KIF6 *and CHD or CVD risk.

	Events/Total			Risk allele frequency	Hazard ratios		
		
	Arg/Arg	Arg/Trp	Trp/Trp		Arg/Arg	Arg/Trp	Arg carriers
CARE[6]^1^	16/155	82/636	44/542	35.5	1.33 (P = 0.33)	1.54 (P = 0.02)	1.50 (P = 0.03)

WOSCOPS[6]^2^	35/94	137/341	104/360	33.3	1.48 (P = 0.11)	1.56 (P = 0.006)	1.55 (P = 0.005)

ARIC [13]^3^	144/1252	474/4363	382/3926	36.0	1.22 (P = 0.03)	1.12 (P = 0.09)	(P = 0.02)^8^

Woman's Health Study [11]^4^	95/3249	349/11831	256/10203	36.3	1.25 (P = 0.09)	1.23 (P = 0.02)	1.24 (P = 0.01)

PROSPER with vascular disease [8]^5^	25/159	119/573	83/514	35.8	1.02 (P = 0.95)	1.36 (P = 0.03)	1.28 (P = 0.07)

PROSPER without vascular disease [8]^5^	13/209	70/759	69/668	36.0	0.64 (P = 0.15)	0.87 (P = 0.43)	0.82 (P = 0.23)

Heart Protection Study [9]^6^							1.17 (P = 0.2)

CHS-White [12]^7^							1.29 (P = 0.005)

CHS-Black [12]^7^							4.14 (P = 0.08)

^1 ^Secondary prevention of fatal and nonfatal myocardial infarction; ^2^Primary prevention of CHD death, nonfatal myocardial infarction, revascularization; ^3 ^Primary definite, probable and silent myocardial infarctions, definite fatal CHD death, or coronary revascularization; ^4^Primary myocardial infarction or stroke, coronary revascularization, cardiovascular death; ^5^Primary or secondary prevention of CHD death, myocardial infarction, or revascularization; ^6^Major coronary events; ^7^Primary or secondary myocardial infarction (definite or probable nonfatal and definite fatal). ^8^Additive model.

Recently, Assimes et al. reported that KIF6 719Arg carriers showed no greater odds for coronary artery disease when 17,000 cases were compared to 39,369 controls of European descent in a meta-analysis of 19 case-control studies [15]. The studies included myocardial infarctions, and clinically significant coronary atherosclerosis without myocardial infarction including ischemia, unstable angina, and revascularization procedures. The meta-analyses included studies whose subjects were enrolled weeks, months and years after their initial CAD event. There was no adjustment for statin use or traditional risk factors other than age and sex. The absence of a significant risk increase for KIF6719Arg carriers was reported for both the total sample and for early onset cases and matched controls, and appeared consistent across individual studies.

This report presents computer simulations to assess the impact of survival bias and effect measure modification by statin use on the detection of genotype-phenotype association in case-control studies. There is a 50% greater risk for recurrent myocardial infarction in carriers of the *KIF6 *719Arg variant, which suggests poorer survival for this genotype [6]. These simulations focus on the *KIF6 *Trp719Arg polymorphism due to its having shown strong, repeated associations with CHD prospectively [6-8,11-14], however, survival bias and effect-measure modification may also cause difficulties in identifying and replicating other CHD-related genetic polymorphisms in cross-sectional case control-studies.

## Results

### Survival bias

**Figure 1:**
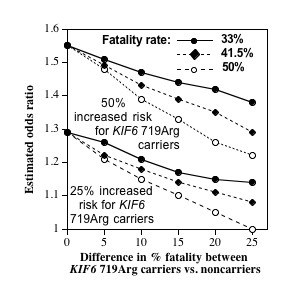
**Simulated effects of genotype-differences in fatalities on the odds ratio for CVD in *KIF6 *719Arg carriers vs. noncarriers**. *KIF6 *719Arg carriers are assumed to represent 59% of the population and to increase CVD risk by 50% (consistent with WOSCOPS and CARE trials [6]) and by 25% (consistent with ARIC and WHS [11,13]) relative to noncarriers. X-axis represents the percent difference in fatality between carriers and noncarriers of the *KIF6 *allele (see methods).

Figure 1 demonstrates the potential bias arising from subject exclusion due to prior fatalities. Results are presented for 33%, 41.5%, and 50% exclusion due to prior fatalities among incident CVDs. That is, 50% exclusion means that for the population from which the cases are drawn, 50% of the potential cases died and are not available to be chosen as cases in a cross-sectional comparison. The X-axis represents the difference in fatalities between genotypes. That is, zero on the X-axis indicates that even though *KIF6 *719Arg carriers are more likely to have a myocardial infarction than is a noncarrier, given that a myocardial infarction has occurred, a *KIF6 *719Arg carrier is no more likely to die from the event than is a noncarrier. In the absence of any difference in percent fatalities between genotypes, odds ratios of 1.55 (for 50% increased risk for the *KIF6 *719Arg carriers) and 1.29 (for 25% increased risk for carriers) were obtained regardless of the portion that survived, otherwise the odds ratios were increasingly attenuated as the disparity in fatality rates between genotypes increased. For example, assuming a 25% increased risk for KIF6 719Agr carriers vs. noncarriers and an overall fatality rate of 41.5%, the odds ratio was reduced to 1.18, 1.11, and 1.08, respectively, for 10%, 20%, and 25% differences in %fatality between carriers and noncarriers. The attenuation increases substantially with the fatality rate when the rate was disproportionate between genotypes (e.g., the odds ratios at 33%, 41.5% and 50% showed no difference when there was no difference in %fatalities between carriers and noncarriers, showed moderate differences when there was a 10% difference in %fatalities between carriers and noncarriers, and large differences when there was a 25% difference in %fatalities between carriers and noncarriers.

### Bias due to the differential effect of statin treatment by KIF6 719Arg carrier status

**Figure 2:**
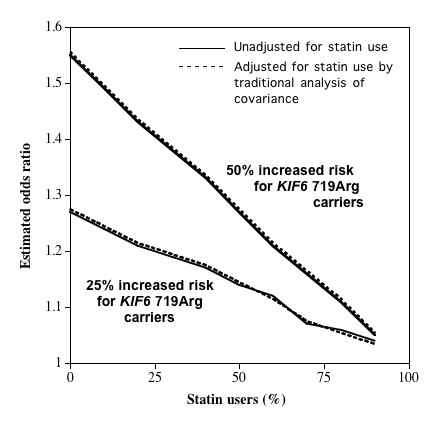
**Simulated effects of statin use on the odds ratio for CHD in *KIF6 *719Arg carriers vs. noncarriers**. Results are presented where *KIF6 *719Arg carriers have 50% or 25% increased risk of CHD compared with noncarriers if there is no statin use, and 0% increased risk of CHD compared with noncarriers if statins are used. Odds ratios (vertical axis) are presented for increasing percentage of statin use (horizontal axis).

The analyses of Figure 2, assume that *KIF6 *719Arg increased CVD risks by 50% (consistent with WOSCOPS and CARE trials [6]) and by 25% (consistent with ARIC and WHS [11,14]) in nonstatin users, and by 0% in statin users. Without statin use, the *KIF6 *719Arg carrier vs. noncarrier odds ratios were 1.55 and 1.27, respectively. The odds ratios decline with greater statin use, i.e., at 50% and 25% increased risk for KIF6 719Arg carriers vs. noncarriers, the odds ratios declined to 1.43 and 1.21 with 20% statin use, respectively, declined to 1.33 and 1.17 with 40% statin use, respectively, and declined to 1.21 and 1.12 for 60% statin use, respectively. By 90% statin use the odds ratio for CVD was essentially one for KIF6 719Arg carriers compared with noncarriers.

A second important result of Figure 2 is that standard statistical adjustment for statin use did not correct for the drug's attenuation of CVD risk in KIF6 719Arg carriers. Identical curves were obtained whether or not statin use was included in the logistic regression model as a covariate. This is because statin interacts with the KIF6 719Arg carriers in determining CVD risk, rather than contributing additively to the log odds for CVD.

**Figure 3:**
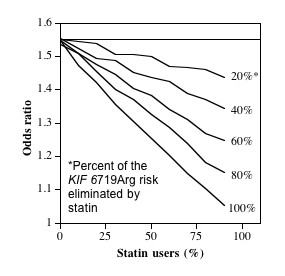
**Simulated effects of statin use on the odds ratio for CHD in *KIF6 *719Arg carriers vs. noncarriers**. for varying effects of statin on *KIF6 *719Arg carriers risk. Results are presented where *KIF6 *719Arg carriers have 50% increased risk of CHD compared with noncarriers if there is no statin use, and where statin use eliminates 20%, 40%, 60%, 80% and 100% of the excess risk associated with *KIF6 *719Arg carrier status (Figure 2 assumed 100% of the excess risk was eliminated by statin use). Odds ratios (vertical axis) are presented for increasing percentage of statin use (horizontal axis).

Whereas Figure 2 assumes that all of the excess risk associated with KIF6 719Arg carrier status is eliminated by statin use, Figure 3 examines the effect of varying the risk reduction associated with statin. Figure 3 suggests that the odds reduction is proportional to the percentage of the excess risk eliminated by statin use.

## Discussion

In prospective cohort studies, genetic variants are identically recruited at baseline and ascertained for incident diseases during follow-up using standardized diagnoses. In case-control studies, spurious associations between genotype and phenotype may arise when the study design or subject participation leads to genotypic differences between cases and controls that are unrelated to the etiology of the disease itself. Responder bias is potentially greater for case-control than cohort studies because recruitment or response may differ between cases and controls. For example, it is not uncommon for control subjects to be derived from geographical, occupational, or environmental sources that differ from those for cases [16]. Cases may also be more motivated to participate than controls in studies that are relevant to the cases' own health concerns. The general population may not identify as strongly with the study goals as do cases, and those that volunteer may be healthier than the general population.

In their review of psychiatric case-control studies, Lee et al. concluded, "Genetic studies achieved the poorest ratings in reducing selection bias" [17]. They point out that few case-control studies adequately describe participant recruitment, which limits their evaluation and potential for replication. Genetic case-control studies require very large samples sizes in order to identify associations between SNP and disease at genome-wide statistical significance, or to obtain precise estimates of the SNPs phenotypic effect. This may require the inclusion of case-control comparisons that are poorly matched. For example, 10 of the 19 different case-control comparisons presented by Assimes et al. had nearly twice as many females in the controls than the treatment groups (including one comparison of exclusively male cases vs. a control group of over 50% women) [15]. This may not affect the analyses because effects of *KIF6 *719Arg carrier are not known to associate with either sex or age, however, CHD is strongly age and sex dependent and the analyses does rely on the adequacy of the statistical adjustment to correct for their effect.

### Survival bias

Our computer simulations show that survival bias, a type of incidence-prevalence bias [18], can substantially attenuate the underlying risk associated with genetic polymorphisms in case-control studies. This occurs when there is greater exclusion of cases in high-risk genotypes due to reduced survival. For a fixed sample size, the decline in the odds will correspond to a reduction in statistical significance, although even very small risk reductions will remain statistically significant if the sample size is sufficient. In contrast, prospective cohort and nested control studies include all incident cases during follow-up, including fatal cases. Others have discussed survival bias in non-genetic context, for example with regards to the interpretation of trauma treatment [19]. Although generally recognized in epidemiological research, survival bias remains a largely unrecognized problem of gene-disease associations in genetic cross-sectional case-control studies.

### Bias due to the differential effect of statin treatment by KIF6 719Arg carrier status

In addition to survival bias, failure to account for statin use may contribute to the weaker association between *KIF6 *genotypes and CHD in case-control studies. Four studies have thus far demonstrated that statins significantly reduce CHD-risk in carriers of *KIF6 *719Arg, whereas noncarriers derive less benefit from statins [6,8,10]. This disparity in statin benefit will attenuate the association between incident CHD and *KIF6 *719Arg in studies that fail to exclude statin users. For example, Figure 2 suggests a weaker association between the *KIF6 *719Arg and CHD in several studies included in the analyses by Assimes et al. [15] might be due, in part, to statin use among cases (e.g., case use of lipid lowering drugs were reported for 74% in the WTCCC study [20], 66% in the German Myocardial Infarction Family Studies [20], 62% in the Heart Attack Risk in Puget Sound Study [21], 35% in the Massachusetts General Hospital Premature CAD Study [22]) in addition to survival selection. The Ottawa Heart Genomics Study [23] also found no difference in the *KIF6 *719Arg allele distribution between angiographically defined coronary artery disease cases and community controls, which could be due, in part, by high statin use (89%) among the cases, in addition to survival selection. From the descriptions provided, the portion of the statin users that were on their medication prior to their myocardial infarction or angiographically defined coronary artery disease cannot be determined for Ottawa Heart Genomics Studies and the various studies used by Assimes et al. The statin usages cited above would include more aggressive lipid interventions subsequent to the event, and post-incident statin use may actually prevent the loss of *KIF6 *719Arg carriers among cases.

The nonsignificance of the Ottawa Heart Genomics Study may also reflect in part an effect of the *KIF6 *genotypes on CHD that is independent of the extent of atherosclerosis [23]. CHD risk reductions in statin-treated *KIF6 *719Arg carriers appear to occur too soon to be attributable to reduced stenosis and may relate to improved plaque stability [10]. Two studies, in fact, show statins reduced CHD events in some individuals experiencing little LDL-C lowering [8,10]. In this regard, it is not surprising that two studies that designated cases by angiography scores in Assimes et al.'s analyses showed no increased risk for *KIF6 *719Arg carriers [24,25].

The potential effect-measure modification due to statin use is not limited to case-control studies. Three of the prospective studies of Table 1 did not explicitly exclude or control for statin use, which may have attenuated or eliminated *KIF6 *719Arg's true risk. The ARIC study showed that homozygotes and heterozygotes of the *KIF6 *719Arg risk allele had 22% and 12% greater risk for incident CHD, respectively, when adjusted for age and sex [13] (P = 0.05 for additive model). The CHS 13-year follow-up of 3,849 white men and women aged 65 years and older showed that *KIF6 *719Arg increased the risk of incident myocardial infarction by 29% [12]. The WHS 12-year prospective follow-up of 26,274 initially healthy women ≥45 years showed that carriers of the *KIF6 *719Arg had 34% greater risk for myocardial infarction and 24% greater risk for total cardiovascular events (cardiovascular death, myocardial infarction, ischemic stroke, and revascularization procedures) than noncarriers [11].

Historically, candidate genes have been more prone to false positives than GWA studies [26], presumably because little attention was given to the total number of candidate SNPs targeted in these initial studies, and not because there were biological rationales for SNP selection. This phenomenon is not relevant to *KIF6 *719Arg results that have been shown to be significant in 6 out of 9 study groups, and positive in 8 out of 9 study groups (Table 1), i.e., if the initial discovery of increased risk for *KIF6 *719Arg had been a false positive, then we would expect only 5% of the subsequent study groups would show significantly increased risk. Historically, poor reproducibility was also true for GWAS discoveries that failed to adequately correct for multiple hypothesis testing. Of course, GWAs can also be applied to prospective studies.

The attenuating effects of statin on *KIF6 *719Arg's association with CHD are akin to the attenuating effects of folate on MTHFR (5,10-Methylenetetrahydrofolate)'s association with CHD. Those having the 677C_T (Ala222Val) substitution in the MTHFR gene have lower MTHFR and higher plasma homocysteine levels [27-29]. Whereas some studies report an association between MTHFR and CHD or myocardial infarction [30-34], others do not [29,35], which has been attributed to an amelioration of homocysteine risk by folate and B vitamins. Higher folate intake has also been proposed to explain why the 677C→T (Ala222Val) polymorphism of *MTHFR *predicts CHD in the Middle East and Asia where folate intake is low, but not in Europe, North America, or Australia where folate intake is high [36]. A cautionary note of treatment associated shrinkage of risk effects was addressed statistically by Tobin et al. in context of anti-hypertensive therapy and provides further support of the potential underestimation of genetic associations when unadjusted for treatment [37].

### Limitations

Our simulated results should be interpreted with caution. The parameters we set in our simulations to illustrate the potential impact of differential fatality and statin use on the outcome of a case-control genetic association study may be less extreme in different settings, for example there may be a smaller attenuating effect of statin on CVD risk in *KIF6 *719Arg carriers. Although disproportionate CHD survival for *KIF6 *Trip719Arg carriers is suggested by their 50% greater risk for recurrent myocardial infarction, we currently do not have direct evidence for a difference in fatality following cardiovascular events between 719Arg carriers and noncarriers, nor are we aware of other genetic variants that are associated with increased fatality after cardiovascular event. The moderate effect of the *KIF6 *719Arg variant on the risk of cardiovascular events may suggest that differences in fatality following cardiovascular events are small. This could explain a lack of significant deviation from Hardy-Weinberg equilibrium expectation for the *KIF6 *Trp719Arg polymorphism in individuals 65 years or older [12], although the statistical power to detect such differences may be lacking. The prevalence of statin use among those eligible to become cases, prior to their index event may be modest as well. For example, 5% to 15% statin use occurring in populations where cases are drawn in case-control studies of cardiovascular disease, would have a negligible effect on the risk estimate (Figure 2). Finally, our simulations considered each of these parameters independently; however, these factors may interact, in aggregate, to result in shrinkage of higher risks of coronary events.

## Conclusions

In summary, our simulations illustrate several potential pit-falls to genetic case-control studies of disease risk. Discrepancies between prospective and case-control studies are not uncommon, with the hormone replacement therapy being one of the better-known examples. Whereas prior case-control studies of hormone replacement therapy suggested 30% to 50% reductions in CHD risk and little increased risk for stroke [38], its use in the Women's Health Initiative was terminated early due to significant increase stroke risk with little apparent reduction in CHD risk [39]. It seems unlikely that the significant increases in CHD risk in *KIF6 *719Arg carriers is simply a chance occurrence (Table 1), and that careful consideration as to why prospective and case-control designs may lead to differing results is warranted.

Prospective cohort studies may lack the cost savings for studying specific disease outcomes and generally require extended elapsed time to complete, but likely provide less biased results. Cohort studies also produce archival genetic material that can be interrogated afresh to test and verify emerging hypotheses. When based upon case-control studies, where inclusion depends upon the genotype affecting survival, the small to moderate CHD risks heretofore attributed to most polymorphisms may be underestimates of substantially greater actual risk. Further, other genetic variants that are associated with both disease risk and drug response may have been missed in GWA studies where underlying drug use among high-risk cases is often unknown and could be extensive. These concerns regarding genetic associations in cross-sectional case-control study design are in addition to other potential caveats discussed by others [40-42]. The apparent "missing heritability" of genome wide association studies is well recognized (i.e., the heritability difference as estimated from family and twin studies versus GWA studies [43]). In advance of potentially uncovering rare variant associations through genome sequencing studies, whose cost-effectiveness will be delayed for years [44], the survival and treatment biases noted here should be considered as potential factors in the reduction in effect size of associated risk variants of genome-wide association studies [45,46].

## Methods

Simulations were performed using the uniform random number generator and logistic regression statistical analysis of JMP version 5.1 (SAS institute, Cary SC). To assess the impact of survival bias, ten replicates of 1,000,000 observations each were generated based on the age and sex distribution of Canadians from the 2006 census [47]. These were assigned CVD risk from the Framingham Health Study based on their sex and cumulative disease incidence between age 40 and their current age [48]. CVD risk was examined because ischemic stroke was included among endpoints in the WHS [11]. Fifty nine percent of the sample was randomly designated as *KIF6 *719Arg carriers in accordance with published reports [6-8,11-13] and their CVD risk increased by 50% (consistent with WOSCOPS and CARE trials [6]) or by 25% (consistent with ARIC and WHS [11,14]) relative to noncarriers. Events assigned by comparing an individual's CVD risk to random number generated from the uniform distribution, from which we excluded randomly selected cases in accordance with carrier-specific survival rates. The difference in survival rate between KIF6 719Arg carriers and noncarriers was specified to keep the overall rate constant (e.g., by setting the fatality rate to 55% in carriers and 43% in noncarriers, the overall event rate remains 50% in carriers and noncarriers combined, i.e., 55%*.59 + 43%*.41 = 50%, and there is 25% reduction in the event rate for carriers vs. noncarriers, i.e., 43 fatalities per 100 events is 25% less than 55 fatalities per 100 events). Simulated data were then analyzed by logistic regression analyses with CVD as the dependent variable, and age, sex, and *KIF6 *719Arg carrier status as the independent variables. In these simulations, the particular population demographic affects on the disease incidence has little affect on the selection bias (verified by modest changes in the simulation, results not displayed). The Canadian population was chosen to address the potential affects of survival bias for a particular Canadian-based report that failed to demonstrate statistical significance [23]. With 95% confidence, ten replicates of 1,000,000 observations were found to estimate the odds ratio within ±0.02.

To assess the bias due to the differential effect of statin treatment by *KIF6 *719Arg carrier status, ten replicates of 1,000,000 observations were again generated based on the age and sex distribution of Canadians, assigned CVD risk using the Framingham Health Study [47], and 59% of the sample randomly designated as *KIF6 *719Arg carriers. Statin users were chosen at random for 0%, 10%, 20%, ... 90% of the sample. The simulated data were again analyzed by logistic regression analyses with CVD as the dependent variable, and age, sex, and *KIF6 *719Arg carrier status as the independent variables. We also included statin use as an independent variable to assess whether traditional statistical adjustment corrects for statin use.

## Abbreviations

ARIC: Atherosclerosis Risk in Communities study; CARE: Cholesterol and Recurrent Events secondary prevention trial; CHD: coronary heart disease; CHS: Cardiovascular Health Study; CVD: cardiovascular disease; HPS: Heart Protection Study; *KIF6: *kinesin-like protein 6; LDL: Low-density lipoproteins; MTHFR: 5,10-Methylenetetrahydrofolate; PROSPER: Prospective Study of Pravastatin in the Elderly at Risk Study; WHS: Women's Health Study; WOSCOPS: West of Scotland Coronary Prevention Study; WTCCC: Wellcome Trust Case Control Consortium

## Competing interests

HRS and PW have received funds from Celera, whose product could profit from this publication. LP has no financial competing interest to report. Celera paid for the preparation and processing charge for the manuscript.

## Authors' contributions

PW performed the computer simulations and wrote portions of the paper; LP and RS wrote and revised portions of the paper and provided clinical perspectives to the results.

## Pre-publication history

The pre-publication history for this paper can be accessed here:

http://www.biomedcentral.com/1471-2350/12/42/prepub
